# Perspective on the dynamics of cancer

**DOI:** 10.1186/s12976-017-0066-5

**Published:** 2017-10-03

**Authors:** Youcef Derbal

**Affiliations:** Ted Rogers School of Information Technology Management, Ryerson University, Toronto, Canada

**Keywords:** Cancer dynamics, Tumor microenvironment, Cardinal signaling pathways, Cancer inflammation, Tumor immunosuppression, Dysregulated cellular processes, Cancer associated fibroblasts, Tumor associated macrophages

## Abstract

**Background:**

The genetic diversity of cancer and the dynamic interactions between heterogeneous tumor cells, the stroma and immune cells present daunting challenges to the development of effective cancer therapies. Although cancer biology is more understood than ever, this has not translated into therapies that overcome drug resistance, cancer recurrence and metastasis. The future development of effective therapies will require more understanding of the dynamics of homeostatic dysregulation that drives cancer growth and progression.

**Results:**

Cancer dynamics are explored using a model involving genes mediating the regulatory interactions between the signaling and metabolic pathways. The exploration is informed by a proposed genetic dysregulation measure of cellular processes. The analysis of the interaction dynamics between cancer cells, cancer associated fibroblasts, and tumor associate macrophages suggests that the mutual dependence of these cells promotes cancer growth and proliferation. In particular, MTOR and AMPK are hypothesized to be concurrently activated in cancer cells by amino acids recycled from the stroma. This leads to a proliferative growth supported by an upregulated glycolysis and a tricarboxylic acid cycle driven by glutamine sourced from the stroma. In other words, while genetic aberrations ignite carcinogenesis and lead to the dysregulation of key cellular processes, it is postulated that the dysregulation of metabolism locks cancer cells in a state of mutual dependence with the tumor microenvironment and deepens the tumor’s inflammation and immunosuppressive state which perpetuates as a result the growth and proliferation dynamics of cancer.

**Conclusions:**

Cancer therapies should aim for a progressive disruption of the dynamics of interactions between cancer cells and the tumor microenvironment by targeting metabolic dysregulation and inflammation to partially restore tissue homeostasis and turn on the immune cancer kill switch. One potentially effective cancer therapeutic strategy is to induce the reduction of lactate and steer the tumor microenvironment to a state of reduced inflammation so as to enable an effective intervention of the immune system. The translation of this therapeutic approach into treatment regimens would however require more understanding of the adaptive complexity of cancer resulting from the interactions of cancer cells with the tumor microenvironment and the immune system.

**Electronic supplementary material:**

The online version of this article (10.1186/s12976-017-0066-5) contains supplementary material, which is available to authorized users.

## Background

Cancer is a complex disease which continues to challenge old and newly approved therapeutic drugs. The relapse of treated patients and the inevitable drift to metastasis highlight the adaptive complexity of cancer. Although the mechanisms underlying the genesis and progression of cancer are better understood than ever [1], the therapeutic drugs being developed so far did not lead to an inflexion towards a cure for all patients [2]. The collateral damage of chemotherapy and radiation and the inevitable onset of resistance followed by metastasis is a serious limitation of the current cancer armamentarium. The selective targeting of oncogenes through kinase inhibition is promising in its rationale but equally exposed as a therapy to the problem of drug resistance. Combining multiple drugs is an approach that has been explored to overcome resistance, however more research is needed to achieve effective combinations of drugs that are tolerable and non-interactive [3]. Immune checkpoint blockades provide another cancer therapeutic avenue with a clinically proven potential [4–10]. Immunotherapy’s impact on patient survival rate and lifespan will ultimately depend on the extent to which an effective antitumor immunity is achieved with manageable immune toxicity [11]. Adoptive cell transfer using engineered T cells that recognize specific cancer antigens has shown promising clinical results against some cancers such as acute lymphoblastic leukemia [12, 13]. However, given cancer heterogeneity, finding target antigens that are unique to cancerous cells is a critical challenge for this type of therapy [14]. On the other hand, oncolytic virotherapy, which has recently received increased attention, faces the formidable challenge of virus delivery and intratumoral spread and cross-priming the host immune system against the cancer while mitigating safety concerns such as virus mutability and unexpected toxicity [15]. Other cancer therapeutic strategies have also been explored, including epigenetic therapy consisting of DNA-demethylation and inhibition of histone deacetylases to undo the effect of mutated chromatin-remodeling enzymes implicated in cancerous cell proliferation [16–18]. These advances in cancer drug development are increasingly leveraged within integrated treatment strategies, combining surgery, radiation, chemotherapy, endocrine therapy, kinase inhibition and immune checkpoint blockades, to extend their therapeutic reach to larger groups of patients and achieve longer remission periods for those patients that are responsive [19]. Although an improvement of the survival rates for some types of cancer have been achieved, a cure is still beyond reach [2]. Indeed, even the simultaneous combination of multiple targeted therapies is predicted to fail in the presence of a single genetic mutation that is resistant to multiple targeted drugs [20]. Drug-conjugated antibodies may not improve cancer free survival either. For example the combination of Bevacizumab and paclitaxel did not deliver any significant benefit for HER2+ breast cancer [3], highlighting the need for more understanding of how to combine antibodies with traditional chemotherapy and targeted tyrosine kinase inhibitors to minimize toxicity and maximize effectiveness. In any case, the evolving genetic heterogeneity of tumors will remain a serious challenge to the development of an effective cancer therapy through the combination of multiple drugs [19, 21]. This complex adaptive nature of cancer, for which even targeted combination therapies would not address unmet therapeutic needs, constitutes a compelling reason to explore a paradigm shift in the search for a cure. It may be argued that cancer adaptive complexity can only be successfully countered by a likewise adaptive therapeutic strategy. The development of such therapeutic system requires a comprehensive understanding of the integrated working of the drivers underlying the dynamics of homeostatic dysregulation that drives cancer progression. Significant advances have been made on this front, yielding a chronological map of the processes underlying carcinogenesis and the cellular and tissue dynamics driving cancer progression leading to metastasis [1]. Indeed, the explicit framing of cancer dynamics in terms of hallmarks identifies specific windows of therapeutic interventions that can be used to disrupt the obstinate march of cancer. The challenge however, is how to counter the adaptive complexity of cancer dynamics in response to therapy. The genetic diversity within a tumor and across tumors of the same cancer type is a formidable challenge making of the disease a moving target which limits the staying effectiveness of most cancer drugs. The active role of the tumor microenvironment (TME) in the promotion and maintenance of tumor growth adds another dimension to the complexity of cancer dynamics. This makes it an imperative that the search for effective therapies should take in consideration not only the genetic drivers of the disease but also the confluence of their effects in collusion with the TME to promote cancer progression. In this respect, the article explores an understanding of cancer dynamics from the perspective that information and energy are the primary organizing drivers of the adaptive complexity of living organisms [22]. More specifically, cancer dynamics are postulated to be driven by the reciprocal dependence between the dysregulated flow of information channeled by the genetically altered cell signaling networks and the energy production and biomass transformations enacted by a reprogrammed metabolism. Furthermore, it is assumed that the TME represents a necessary catalytic milieu enabling the provision and exchange of growth factors and nutrients required for tumor growth. A key element of this view of cancer dynamics is the role of feedback as a double edge lever of biological regulation. Indeed, on one hand feedback enables robustness of biological processes and the maintenance of cellular and tissue homeostasis [23–26]. However, beyond a certain degree of signaling and metabolic dysregulation, feedback between the signaling pathways, metabolism and the TME may become the mechanistic conduit for exacerbating the drift away from homeostasis and for driving tumor growth. In this respect, do the feedback signals and biomass exchanges between cancer cells (CCs) and the TME carry cancer vulnerabilities that can be therapeutically targeted? If such vulnerabilities do exist, then how can they be leveraged to turn on the cancer kill switch and enlist a decisive intervention of the immune system? These and other questions related to cancer dynamics will be explored in the following sections using simplified models of cellular processes and the tumor microenvironment.

## Genesis of cancer dynamics

**Fig. 1:**
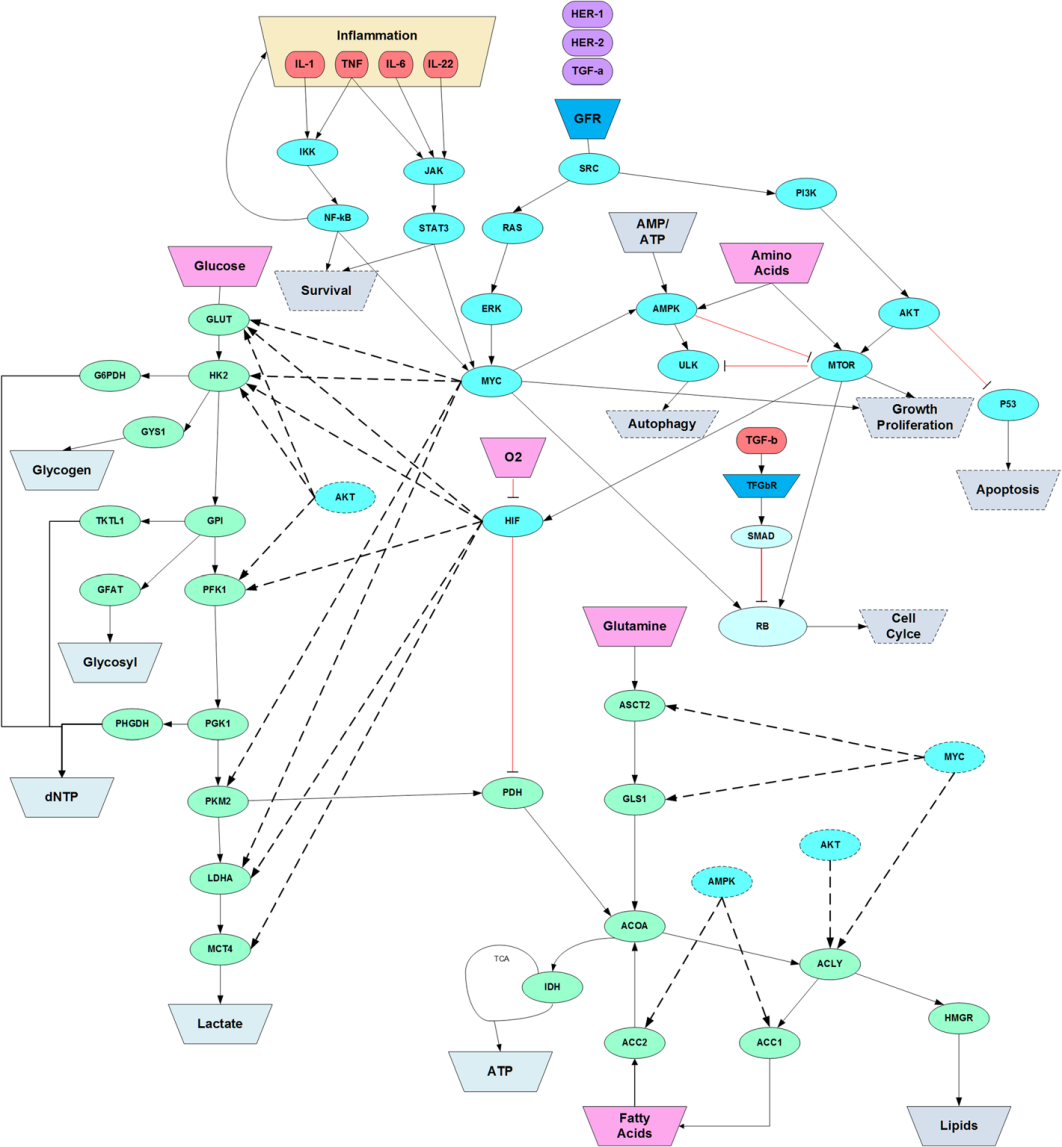
Integrated signaling and metabolic cellular processes. Cellular metabolism is regulated by key signaling pathways of growth and proliferation which include RAS-ERK and PI3K-MTOR. These pathways are also involved in the regulation of the cell cycle, autophagy, survival and apoptosis in concert with the TGFβ, NF-χβ, and P53 signaling pathways among others

The causal effects linking genetic alterations and the phenotypic state trajectories of cancer cells are enacted within the TME context and channeled through operational deviations from homeostasis of growth, proliferation, autophagy, angiogenesis, apoptosis, survival, focal adhesion, cell cycle, DNA repair, and energy production. The dysregulation of these cellular processes are known to implicate various sets of genetic drivers as supported by genome wide studies of different cancers [27–32]. However, the high number of assumed cancer driver genes poses a challenge to the development of a much needed insight about the dynamics of cell signaling and metabolic interactions underlying tissue homeostasis. Furthermore, it is not clear whether all the genes identified by the various genome wide studies have an equally determinant impact on carcinogenesis and cancer progression. Assumptions about the existence of principles driving the overall dynamics of cancer as a system of heterogeneous multiplicity of biological parts and modules, may points to a select high confidence set of cancer implicated genes as the key determinants of carcinogenesis. In this respect, it has been suggested that biological complexity is driven by a reciprocal causality between energy/biomass production and information flow [22]. This notion has been recently supported by a comprehensive analysis of the reciprocal regulation shown to exist between the cell signaling network and the metabolic circuitry [33–35]. Therefore, it may be plausible to hypothesize that the genes mediating the interactions between cell signaling and metabolism are critical determinants of the dynamics underlying tissue homeostasis. These levers of the signaling-metabolic interface include the energy sensor AMPK, the proliferation regulator MTOR, the growth regulators MYC and AKT, the oxygen sensor HIF, and the apoptotic trigger P53 (see Fig. 1). These genes are interconnection hubs of the signaling circuitry that maintains tissue homeostasis and prevents runaway growth and proliferation. For instance, MYC and AKT, which are the end effectors of mitogenic pathways, regulate the uptake of glucose through GLUT as well as the catalytic capacity of the downstream glycolytic enzymes including LDHA and MCT4. An upregulation of mitogenic pathways, whether caused by mutant genes or overabundance of growth factors, would amplify the glycolytic flux feeding the glycosyl pathway, the PPP (pentose phosphate pathway), the serine pathway, and the one carbon metabolism, which drive biomass production, including nucleotides, glycosyl, glycogen, and non-essential amino acids (AA). Under hypoxic conditions, an elevated glycolytic activity can be further enhanced by HIF through its excitatory action on the lactate transporter MCT4 as well as the inhibition of PDH which limits as a result the flux of pyruvate from glycolysis to the tricarboxylic acid (TCA) cycle. Furthermore, the mutant form of IDH found in many cancers leads to the production of the oncometabolite 2-HG which further drives the action of HIF as a promoter of heightened glycolysis under hypoxic conditions. Taken together, MYC and AKT as the glycolytic effectors of the RAS-ERK and PI3K-AKT pathways, can intensify glycolysis either in response to higher levels of extracellular stimuli such as growth factors and cytokines or as a result of oncogenic alterations involving genes such as RAS, RAF and EGFR. Moreover, tumor growth leads to hypoxic conditions in the region trailing the invasive front, causing HIF to deepen the elevated glycolytic regime as explained above. In addition to its regulatory control on glycolytic rate, MYC can upregulate the uptake of glutamine through ASCT2 and its transformation by GLS1 to feed the TCA cycle as well as modulate the synthesis of lipids, with the help of AKT. In summary, the signaling pathways converging on MYC, AKT and HIF have the capacity to reprogram cell metabolism to fulfill the biomass needs of tumor growth by directing the biosynthesis of proteins, lipids and nucleotides while maintaining an adequate level of cellular ATP. However, in order to sustain tumor growth progression, MTOR needs to be coopted to promote runaway cell proliferation by driving ribosomal protein synthesis and translation. While it is widely accepted that MTOR is inhibited by AMPK under conditions of lower cellular energy sensed by a higher AMP/ATP ratio, it has been recently shown that both AMPK and MTOR can be concurrently activated by amino acids [36]. The effects of concurrent AMPK and MTOR activation would constitute a convergence of the cell regulatory dynamics in support of proliferation by driving mitochondrial biogenesis, ATP generation, fatty acid oxidation, ribosomal protein synthesis, translation, cell cycle progression through the restriction point, and autophagy. The drive towards uncontrolled proliferation is further accentuated in the presence of mutant P53 and PTEN since these are expected to be less effective in dampening the survival signals sourced from AKT. The signaling dynamics integrated through the actions of the effectors MYC, AMPK, AKT, MTOR, HIF, PTEN, and P53 can sustain tumor growth progression provided that extracellular stimuli, such as growth factors and cytokines, are maintained along with sufficient availability of glucose, glutamine, amino acids and fatty acids. In other words, while genetic alterations lead to dysregulated signaling and a reprograming of metabolism in support of cell growth and proliferation, synergetic intercellular interactions and a promoting tumor microenvironment will still be required to sustain tumor growth. Of particular interest is the contributions of CAFs (cancer associated fibroblasts) and TAMs (tumor associated macrophages) in helping cancer cells acquire the necessary supply of glutamine, fatty acids, and amino acids on one hand and at the same time maintaining an inflammatory and immunosuppressive environment that protects tumor growth from the intervention of the immune system.

## Role of the tumor microenvironment

**Fig. 2:**
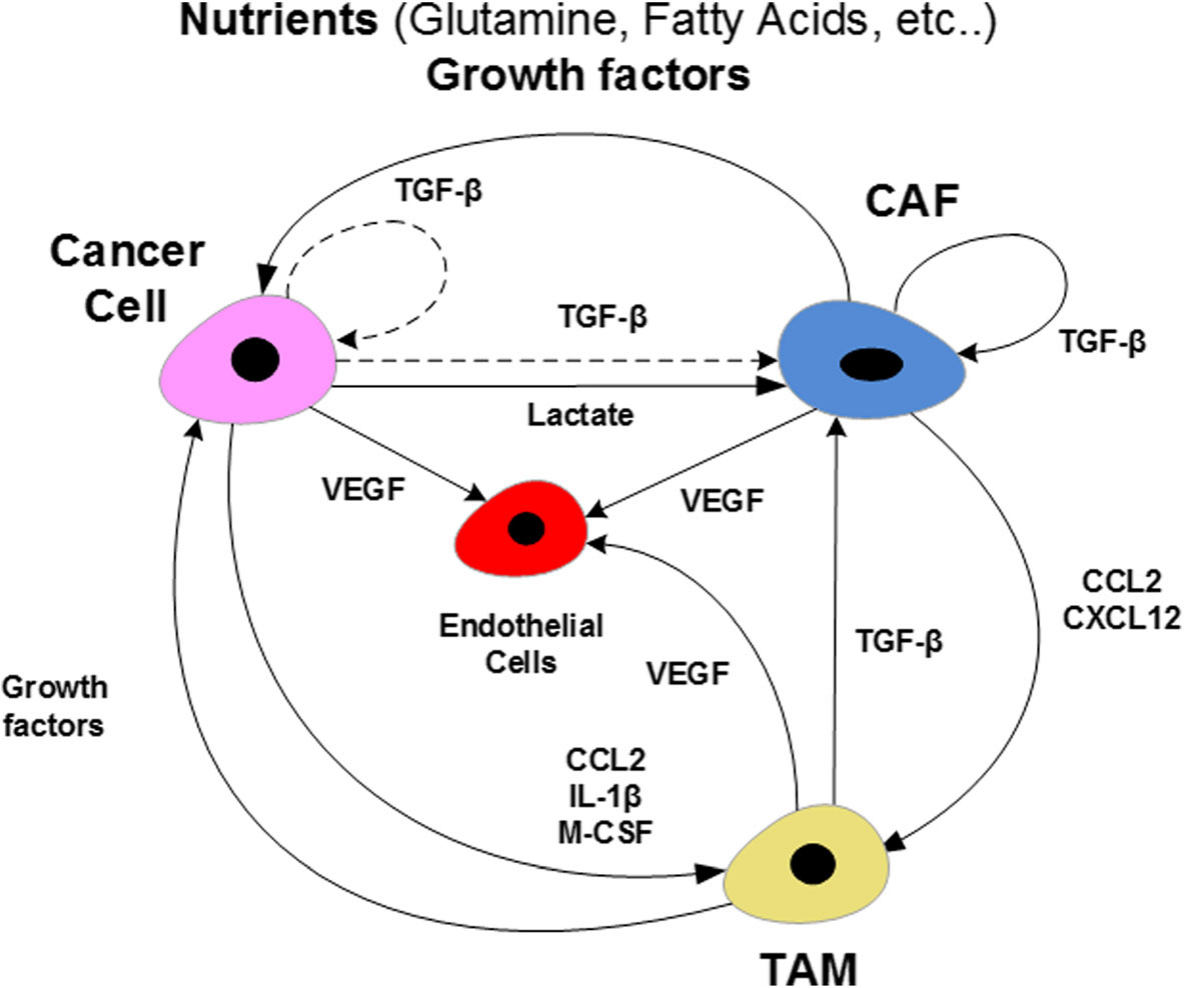
TME Cells’ interactions. The synergetic interactions between CAFs, TAMs and cancer cells promote inflammation, immunosuppression and tumor growth. The dotted lines show indirect interactions, as is the case with the release of TGF-β from the extracellular matrix (ECM) degraded by the actions of proteases secreted by cancer cells

The active role of the TME in promoting and sustaining tumor growth is increasingly accepted as pivotal to cancer progression [37–43]. In particular, it is believed that TAMs and CAFs affect tumor growth and modulate the intervention of the immune system through synergetic interactions with cancer cells [37–39, 44, 45]. More specifically, cancer cells’ secreted cytokines and chemokines, such as TGF-β, are known to activate CAFs [37, 38]. In return, CAFs provide the TME and cancer cells with recycled nutrients such as glutamine and amino acids, believed to be resulting from autophagy caused by oxidative stress, itself induced by adjacent cancer cells [42, 43]. It has also been reported that CAFs and cancer cells co-reprogram their metabolism whereby the lactate output of CAFs feeds the so-called reverse Warburg effect in cancer cells to drive their aerobic metabolism [46]. Furthermore, cytokines and chemokines, such as CCL2, which are secreted by cancer cells and CAFs are known to be involved in the recruitment of macrophages and the induction of their transformation into TAMs [47–49]. Once recruited to the tumor, both TAMs and CAFs have a direct impact on cancer proliferation and metastasis [47, 50]. In particular, CAFs, which supply cancer cells with recycled nutrients and growth factors as discussed earlier, also release TGF-β and promote as a result the immunosuppressive milieu of the TME [51, 52]. The inflammatory and immunosuppressive state of the tumor microenvironment is further reinforced by TAMs, hence shielding cancer cells from the actions of the adaptive immune system [40, 45]. The reciprocal effects characterizing the interactions between TAMs, CAFs and cancer cells will be explored further using the simplified model of TME illustrated in Fig. 2 [1, 37]. The disruption of the interaction signals and nutrient flows between cancer cells, CAFs and TAMs may constitute an effective therapeutic approach to impede the malignant dynamics of the TME and blunt the ability of cancer cells to enlist the support of TAMs and CAFs. Indeed, in addition to the consideration of cancer genetic signatures, cancer therapies should account for the active involvement of the TME in shaping the trajectories of tumor growth dynamics. Therapeutic interventions based on an understanding of these dynamics may lead to desirable clinical outcomes provided that they can shunt the rewiring of the signaling and metabolic networks associated with the accumulation of genetic mutational burden. One step towards the exploration of therapeutic strategies that factor in cancer dynamics would entail understanding how the dysregulated dynamics of the cell signaling and metabolic pathways both impact and reflect the interactions between cancer cells, CAFs and TAMs. Seeking such understanding, a putative model of TME cell interactions, illustrated in Fig. 2, will be used to explore questions about the potential fate of cancer cells under various therapeutic approaches targeting the communication signals and nutrient flows facilitated by the TME. One particular question of interest is: which therapeutic disruption of cancer dynamics would most probably lead to a sustained reversal of tumor growth and keeps at bay the reactionary robustness of the disease state? In other words, is there a kill switch that can be flipped to disable the unholy union between cancer cells, CAFs and TAMs and restore tissue homeostasis?

## Modeling cancer dynamics

Although cancer cell signaling and metabolic dysregulation may be caused by somatic gene mutations, gene copy number variations and DNA hypermethylation, the focus will be primarily on somatic mutations as the main drivers of carcinogenesis. In particular, let *p*_*k*_ , *k* = 1 , … , *N*, N > 0, be the probability that the kth gene in a given pathway *w* harbors deleterious mutations or is subject to copy number variations. The probability *Q*_*w*_ that such pathway is dysregulated is then defined as follows:

1 $$ {Q}_w=1-{\prod}_{k=1}^N\left(1-{p}_k\right) $$

**Fig. 3:**
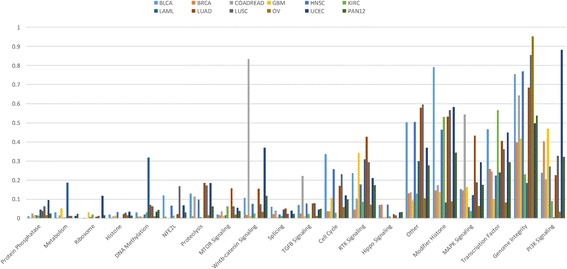
Dysregulation probabilities of cellular pathways. The dysregulation probabilities clearly distinguish the different cancer types and points to a significant differential in the likelihood of altered regulation across different cellular processes. The probabilities are computed using the set of 127 genes and relevant data reported in [27] (Additional file 1: Table S1)

**Fig. 4:**
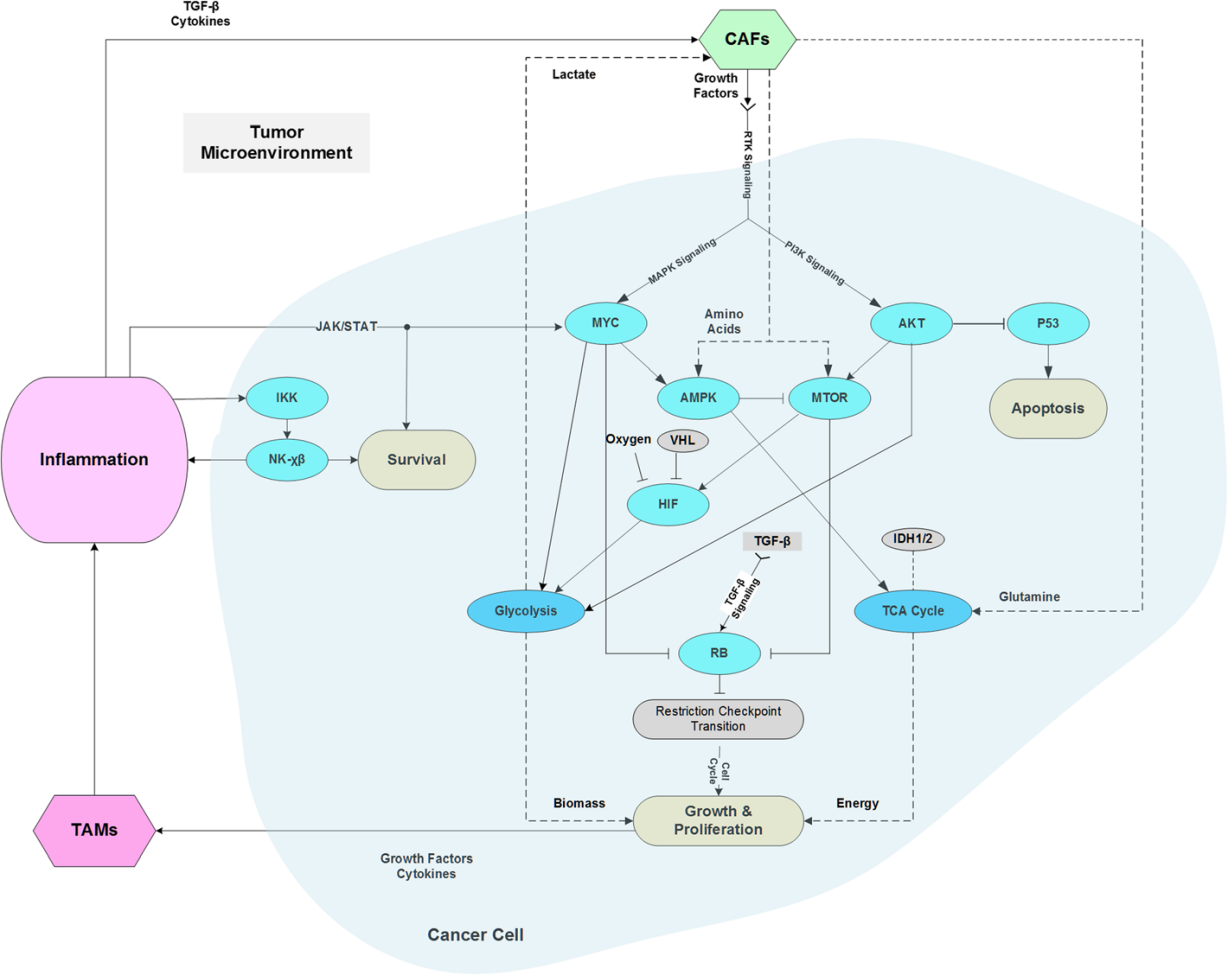
Cardinal pathways mediating the TME-CCs interactions. The TME-CCs interaction dynamics are postulated to be driven by cancer cells enlisting of CAFs and TAMs and the subsequent induction of an inflammation and growth promoting tumor microenvironment

The value of *p*_*k*_ is estimated to be the mutation rate of the gene in question. Using this definition, the probabilities of pathway dysregulation is illustrated for different cancers in Fig. 3 (Additional file 1: Table S1), using the 127 gene set and the classification of cellular processes identified in Kandoth et al. [27]. The likelihood of dysregulation of cellular processes shows a significant dispersion or spread across cancer types as asserted by the corresponding values of the mean and standard deviation of the pathway dysregulation measure (Additional file 1: Table S1). This reiterates the fact that the likelihood of dysregulation for the major cell signaling pathways is dependent on the cancer type. Such variability of the probability of pathway dysregulation as a function of the cancer type also applies to the cell cycle, genome integrity, survival, apoptosis, growth, and proliferation (Additional file 1: Table S1). The “Other” category registers, expectedly, a significant likelihood of dysregulation since it includes genes such as NOCH1, NAV3, MALAT1, and ARHGAP35 known to be associated with cell proliferation as well as other genes such as NPM1 and POLQ which are involved in maintaining genome integrity. While being reductionist compared to the results yielded by the many comprehensive genome wide studies of cancer [27, 28, 30, 31, 53], the proposed measure of pathway dysregulation can be instrumental in the analysis of the interactions between cancer cells and the tumor microenvironment (see Fig.4). Of particular interest is how the effects of these interactions collude with pathway dysregulations to stimulate the dynamics of cancer growth. A number of hypotheses can be put forth about the potential causal chains linking the dysregulation of signaling and metabolic pathways and the initiation and maintenance of cancer growth. First, the probabilities of dysregulation of the RTK, PI3K and MAPK signaling pathways are significantly high for most types of cancer. Driven by growth factors from the stroma, these pathways may, with high probability, be the first drivers of an upregulated glycolysis in cancer cells. The consequent increase of lactate secretion into the TME will thereafter lead to its acidification and the activation of TGF-β [54], leading to the recruitment and transformation of CAFs. In addition to taking up lactate to feed their metabolism, CAFs are thought to undergo autophagy due to oxidative stress induced by cancer cells [37], supplying as a result recycled nutrients such as glutamine and amino acids to neighboring cancer cells. Given the recently reported evidence that AMPK and MTOR can indeed be concurrently activated by amino acids [36], we postulate that it is precisely this additional feedback action of amino acids’ provision by CAFs that stabilizes the initiation of cancer cell growth and proliferation. With the concurrent activation of AMPK and MTOR in cancer cells, the ribosomal protein synthesis and translation processes are activated along with an operational TCA cycle, putatively fed by beta oxidation of fatty acids and recycled glutamine from CAFs. In addition, AKT being the end-effector of the PI3K signaling pathway would facilitate lipid synthesis through its action on ACLY. The CCs-CAFs interactions lead to a dependence between cancer cells and the stroma, whereby cancer cells provide lactate and induce the activation of TGF-β while CAFs provide glutamine, amino acids, fatty acids and growth factors to feed cancer growth. The dynamics of this CCs-CAFs system are further stabilized and perpetuated by inflammation as well as a ratcheted up release and activation of TGF-β in the TME. Furthermore, the oncogenic dysregulation of the RAS, MYC and the MAPK pathways in cancer cells are known to induce the production of growth factors and cytokines such as VEGF, IL-6, IL-10, and IL-1β, leading to the recruitment and the tumorigenic transformation of macrophages [44, 55, 56]. The maintenance of an inflammatory TME is further stabilized through the JAK/STAT and the IKK/NF- χβ pathways, whose effects are robustly sustained by a feedback from cancer cells through the production of inflammatory cytokines such as IL-1β, IL-6 and TNF-α [57–61]. Not only does inflammation feed the accelerated growth and proliferation through the JAK/STAT pathway, it also drives survival through the IKK/NF- χβ pathway (see Fig. 4).

The stability of the CCs-TME dynamics, as described above, deepens the state of inflammation in the TME, whereby cancer growth and proliferation increases the release and activation of TGF-β as well as the secretion of inflammatory cytokines and growth factors maintaining as a result the active roles of the CAFs and TAMs in supporting a cancer promoting TME. The ensuing tumor growth progression is known to be correlated with an increased release and activation of TGF-β in the TME as well as a switch of its role from being a tumor suppressor to a tumor promoter [62–67]. This role switching has been hypothesized to be the result of the balance between the dual, antagonistic effects of TGF-β on cell proliferation induced through its SMAD-dependent and non-SMAD-dependent signaling pathways [63, 64]. In line with this hypothesis, the effects of TGF-β abundance in the TME, channeled through the MAPK and PI3K signaling pathways (see Fig. 4), would further promote the cancer proliferation dynamics driven by the CCs-CAFs-TAMs interactions. At the same time the TGF-β regulation, via its canonical pathway, of the cell cycle passage through the restriction point may be abrogated due to the dysfunction of the RB tumor suppressor (see Fig. 4). These cancer proliferation dynamics are expected to persist given the genetically altered apoptotic and DNA repair pathways, and the immunosuppressive state of the TME promoted by the actions of the TGF-β both as an inducer of Tregs and as an antagonist of the immune functions of NK, DC and T cells [68–70]. Moreover, recently published results have provided new evidence about the role of TGF-β, acting cooperatively with VEGF, in maintaining an immunotolerant TME [71].

## The stochastic dynamics of cancer

The core circuitry driving cancer cell state dynamics is suggested to be dynamically wired to balance the production and use of energy and biomass, supporting the imperative of survival and growth. Glycolysis and the TCA cycle represent the two critical cellular processes responsible for carrying out this imperative. Both processes are regulated by competing signals sourced from growth factor stimuli and channeled through the MAPK and PI3K pathways. The balancing act maintaining energy sufficiency and supporting the growth imperative is brokerage by the AMPK antagonistic action on MTOR, while the availability of energy antagonizes AMPK. It is however plausible, as discussed earlier, that the dysregulation of the RTK, MAPK and PI3K pathways can be locked into a pattern of convergent effects that drive the emergence of a stable CCs-CAFs interaction dynamics. This CCs-CAFs system is suggested to be the source of an AA-dependent concurrent activation of AMPK and MTOR and the subsequent loss of the regulated balance between the activity levels of glycolysis and the TCA cycle. The causal chain implicating genetic mutations in the altered information flow sourced from growth factor stimuli and leading to the loss of energy-biomass homeostasis, may be characterized using the likelihood measure of pathway dysregulation, introduced earlier. In particular, let *Q*_*Gly*_, *Q*_*TCA*_, *Q*_*APO*_, *Q*_*SRV*_, and *Q*_*CCP*_ be the probabilities of dysregulation of glycolysis, the TCA cycle, apoptosis, survival and the cell cycle progression through the restriction point respectively. Given the model of Fig. 4, the dysregulation probabilities are estimated as follows:

2 $$ {Q}_{Gly}=1-{Q_S}^{\ast }{\left(1-{p}_{MTOR}\right)}^{\ast }{\left(1-{p}_{HIF}\right)}^{\ast}\left(1-{Q}_{PI3K}\right) $$

3 $$ {Q}_{TCA}=1-{Q_S}^{\ast }{\left(1-{p}_{AMPK}\right)}^{\ast}\left(1-{Q}_{Metabolism}\right) $$

4 $$ {Q}_{APO}={Q}_{GI} $$

5 $$ {Q}_{SRV}=1-{\left(1-{p}_{IKK}\right)}^{\ast }{\left(1-{p}_{FOX}\right)}^{\ast }{\left(1-{Q}_{Other}\right)}^{\ast}\left(1-{Q}_{TF}\right) $$

6 $$ {Q}_{CCP}=1-{Q_S}^{\ast }{\left(1-{p}_{MTOR}\right)}^{\ast }{\left(1-{Q}_{TGF\beta}\right)}^{\ast }{\left(1-{Q}_{Cell Cycle}\right)}^{\ast}\left(1-{Q}_{PI3K}\right) $$

7 $$ {Q}_S={\left(1-{Q}_{RTK}\right)}^{\ast }{\left(1-{Q}_{MAPK}\right)}^{\ast}\left(1-{p}_{MYC}\right) $$

**Fig. 5:**
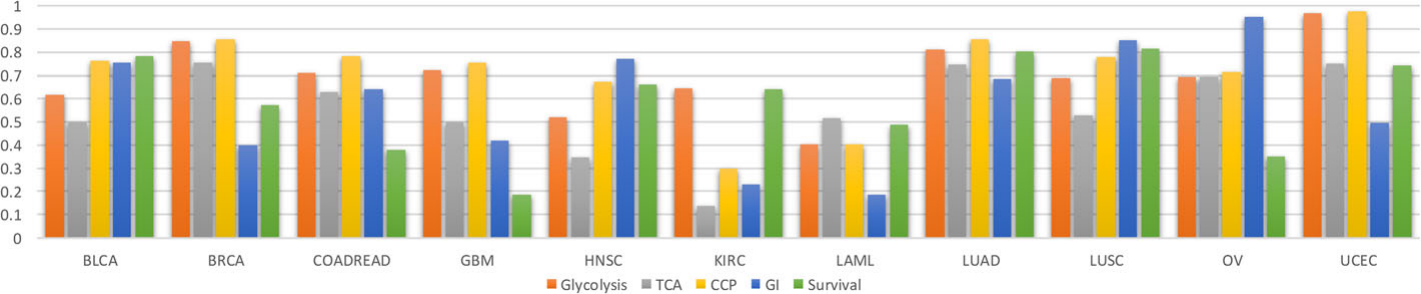
Dysregulation probabilities for glycolysis, the TCA cycle, genome integrity, survival and the cell cycle progression through the restriction point

**Fig. 6:**
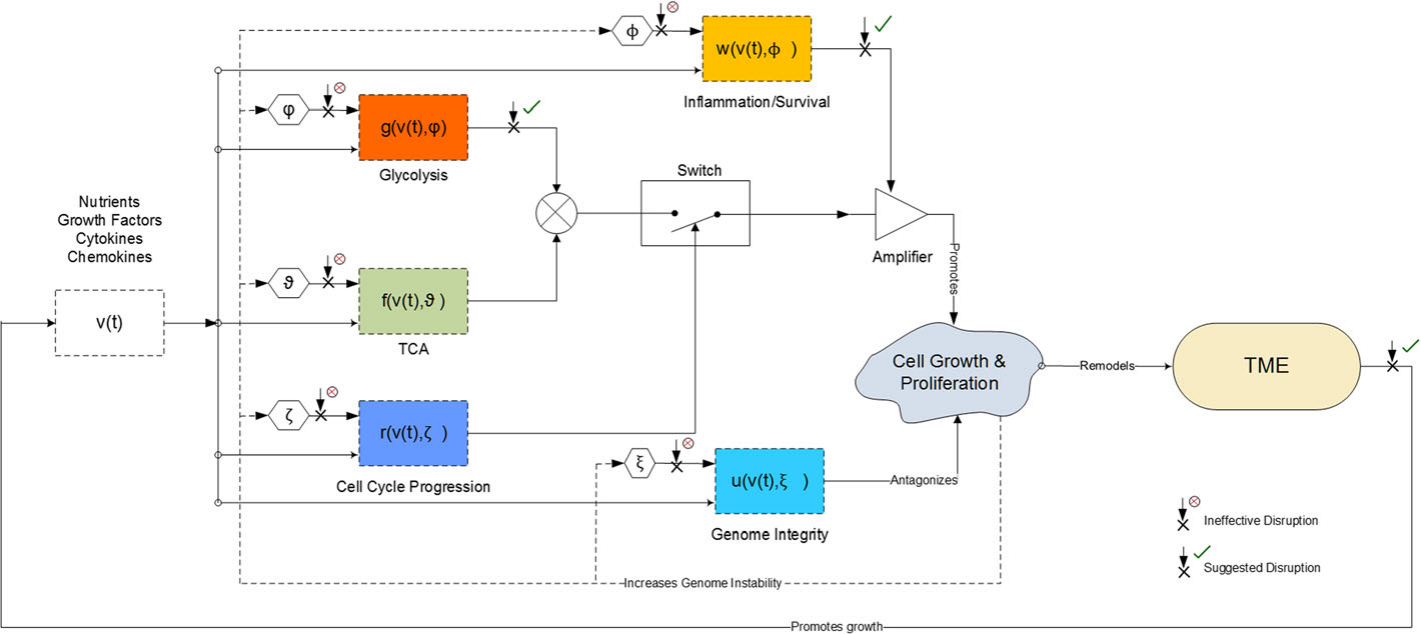
Cancer as a stochastic system. Cancer is postulated to be initiated by the genetic-driven dysregulation of metabolism which is permitted to drive growth and proliferation due to the abrogation of the cell cycle restriction checkpoint and the inflammatory, survival and nutritional feedback of the TME. Therapeutic disruptions of the effectors of cancer growth dynamics are noted and qualified as potentially effective or ineffective based on the level and bandwidth of sensitivity to genomic instability

*p*_*HIF*_ is set to the mutation rate of VHL whose lifted inhibition of HIF leads to the decoupling between glycolysis and the TCA cycle even in the presence of oxygen. Likewise, *p*_*AMPK*_ is set to the cumulative rate of mutation and deletions of LKB1, which activates AMPK in response to ATP depletion relative to AMP and ADP. On the other hand *p*_*MYC*_ is estimated using the cumulative rate of mutation and amplification for the members of the MYC family, in particular MYC, MYCL1 and MYCN (Additional file 1: Table S2). *Q*_*Metabolism*_, *Q*_*GI*_, *Q*_*TF*_, *Q*_*Other*_ are the dysregulation probabilities for the cellular pathways classified in [27] as “Metabolism”, “Genome Integrity”, “Transcription Factors/Regulators” and “Other” respectively. The latter two sets of genes are deemed to be involved either directly or indirectly in the survival pathways along with IKK and the FOX family of genes. The dysregulation probabilities for the five cellular processes (glycolysis, TCA cycle, survival, apoptosis, and cell cycle progression through the restriction point) may constitute a functional signature of the genetic alterations underlying carcinogenesis and tumor growth progression (see Fig. 5, Additional file 1: Table S3). Exploring how the interactions between these key cellular processes lead to the emergence of cancer dynamics may lead to insights about potential vulnerabilities that can be therapeutically targetable. Starting from the assumption that the cell genetic alterations are induced by randomly occurring events, cancer may be viewed as a dynamical system driven by the stochastic states of the cellular processes. Each cellular process *w* can either be in a state of dysregulation with a probability *Q*_*w*_, as computed above, or in a regulated state with a probability 1 − *Q*_*w*_. Let the outputs of glycolysis and the TCA cycle be *f*(*v*(*t*), *ϑ*) and *g*(*v*(*t*), *φ*) representing the residual energy and cell biomass that can be used for growth, where *ϑ* and *φ* are random variables representing the states of glycolysis and the TCA cycle respectively. *v*(*t*) is a vector representing the availability of nutrients, growth factors, and cytokines. The convolution of these stochastic processes defined as $h(t)={\int}_0^tf\left(\tau, \vartheta \right)g\kern0.1em \left(t-\tau, \varphi \right)\kern0.1em d\tau$, where t and τ are time variables, is postulated to represent the cancer initiating signal. In other words, tumorigenic growth is driven by the stochastic convergence of dysregulated TCA and glycolysis processes which leads to the concurrent availability of sufficient energy and biomass to feed a runaway cancer growth and proliferation. The growth signal *h(t)* is subject to the control of the TGFβ pathway which regulates the cell cycle passage through the restriction checkpoint. The regulatory action of the TGFβ pathway is also represented by a stochastic signal *r*(*v*(*t*), *ζ*), where *ζ* is the pathway’s stochastic state. Similarly, the survival and apoptotic signals can also be modeled by stochastic processes denoted as *w*(*v*(*t*), *ϕ*) and *u*(*v*(*t*), *ξ*), where *ϕ* and *ξ* represent the stochastic states of dysregulation associated with the survival and apoptotic pathways respectively (see Fig. 6). This perspective on cancer dynamics places genetic alterations as the initiators of cancer growth through the reprogramming of metabolism and considers this last to be the trigger of the feedback dynamics between cancer cells and the TME. These dynamics will ultimately lead to the removal of the restriction on the cell cycle progression, the amplification of the inflammation sourced survival signals and the emergence of an immunosuppressive TME state. The ensuing tumor growth will further increase genomic instability leading to an accumulation of oncogenic alterations and consequently an increased dysregulation of cellular processes. The model structure assumed to be underlying these stochastic dynamics of cancer may suggest the existence of vulnerabilities that can be leveraged in the design of cancer therapies as well as points to potentially ineffective targets of therapeutic interventions (see Fig. 6). In particular, inhibitions of signaling pathways upstream of the metabolic processes may not be effective because of the potential onset of resistance due to the changing landscape of oncogenic mutations caused by genomic instability. In contrast, therapeutic effectiveness may be achievable by modulating the concentrations of lactate, growth factors and cytokines in the tumor microenvironment (see Fig. 6). Since these concentrations result from CCs-TME dynamics driven by the integration of multiple regulatory signals from the cell and the extracellular environment, they embody, as a result, a smoothed out effect of genetic instability making them more tractable therapeutic targets as will be explored in the next section. However, the metabolic similarity between cancerous tumors and non-cancerous tissues of the muscles and of the nervous system, with respect to the upregulation of glycolysis and the shuttling of lactate, present a toxicity challenge for the clinical success of drugs targeting cancer metabolism, including those aimed at modulating the concentration of lactate in the TME [72, 73]. The availability of comprehensive clinical data sets from the ongoing and planned clinical trials of metabolic inhibitors [72, 74], such as those targeting MCT1, LDHA, and GLUT1, will ultimately enable the clinical assessment of the therapeutic potential of targeting cancer metabolism as proposed in this perspective.

## Is there a cancer kill switch?

Genetic alterations of cellular processes drive the inception of the CCs-CAFs-TAMs interaction system. This induces a progressive ratcheting up of the tumor proliferation dynamics leading to an ever growing genetic heterogeneity and an accumulation of genetic aberrations. The stable persistence of the CCs-TME dynamics enlisting the active involvement of the stroma and inflammatory cells towards cancer growth and proliferation will ultimately lead to invasion and metastasis. Many therapeutic approaches have been explored to target the causal elements believed to be maintaining the CCs-TMEs dynamics. These include the reduction of inflammation, the reduction of lactose excretion by cancer cells and the inhibition of TGF-β ligands [62, 75–82]. In addition, most of the genes and pathways implicated in carcinogenesis have been considered for targeted therapies, including HER/EGFR [83, 84], PI3K-AKT-MTOR [85–87], RAS-RAF-ERK [88, 89], TGF-β [76, 90], AMPK [91–94], the RB pathway [95–97], LDHA [75, 81, 98, 99], MCT1 [100, 101], and NF-χβ/IKK [59, 77, 102]. While the prospect of targeted therapies may be promising [103–110], the specter of acquired disease resistance looms large, representing a persistent challenge to the development of a decisive cancer therapeutic strategy [111–116]. Nevertheless, we speculate that therapies targeting cancer metabolism and TME inflammation might prove effective if combined within a metronomic strategy with the aim to induce a progressive dragging of the CCs-TME dynamics away from tumor promotion and along a staged restoration of tissue homeostasis that avoids the incitement of drug resistance or radical wound repair-like tissue reactions. In particular, given the putative structure underlying the stochastic dynamics of cancer (Fig. 6), and the expected stochastic convergence of dysregulated cellular processes highlighted in Fig. 4, cancer cells must be first denied the glutamine and growth factors lifeline believed to be extended by the stroma in exchange for their secretion of lactate. This could be achieved through the inhibition of LDHA, suggested to be a promising cancer therapeutic target due its role as the catalyst of the pyruvate conversion into lactate, which is subsequently released in the TME [117–120]. Furthermore, reducing the concentration of lactate in the TME would limit cancer cells’ inward uptake of lactate through MCT-1, which was suggested to indirectly increase glutaminolysis by upregulating the expression of the glutamine transporter ASCT2 in a MYC and HIF-2α dependent manner [80]. In addition, a reduction of TME lactate, accepted to be a promoter of inflammation and angiogenesis [75], would not only weakens the TME sourced survival signals driving cancer growth, but it will also disrupt the CCs-CAFs interaction dynamics that are believed to enable a steady supply of recycled nutrients and growth factors from the stroma to the tumor [37]. In fact, we speculate that the lactate-promoted CCs-CAFs interactions and the suggested resulting supply of amino acids to cancer cells by CAFs undergoing autophagy [37], may be among the key inducers of carcinogenic transformations. Indeed, these CCs-CAFs interactions may be the drivers of the concurrent activation of AMPK and MTOR, which was recently reported to be inducible in an amino acid dependent manner [36]. In this respect, targeting the enzymes and metabolic transporters, such as LDHA, MCT1 and GLUT, to limit the production of lactate and its bi-directional shuttling between cancer cells and the TME would not only hinder the reprogramming of metabolism toward unchecked growth but it would also dampen inflammation and angiogenesis. However, targeting cancer metabolism is fraught with challenges associated with the genetic diversity of tumors, their metabolic flexibility and the dose limiting toxicity of metabolic inhibitors due to the fact that metabolic pathways in normal tissue are often equally upregulated just as in tumors [72]. Nevertheless, despite the genetic diversity of tumors and their tissue-specific metabolic reprogramming, the metabolic changes exhibited by different types of cancers are convergent towards an upregulated glycolysis and nucleotide synthesis, a downregulated fatty acid oxidation and a heterogeneous oxidation-phosphorylation [121, 122]. This may justify the exploration of therapies aimed at the metabolic vulnerabilities common across tumor types, in addition to the development of cancer-specific drugs targeting cancer metabolism. Towards this end, the tissue-specific metabolic transformations of tumor cells and their metabolic flexibility, which are due to the heterogeneous signaling and the dynamic distributions of nutrients, oxygen, and catabolites in the tumor microenvironment, need to be better understood and characterized to enable patient stratification based on the metabolic profiles of tumors and to target cancer metabolism accordingly. Overall, limiting the impact of metabolic dysregulation combined with a reduction of inflammation by targeting NF-χβ [77], TNF-α [123], the Jak/Stat pathway [124] and the TGF-β pathway [76] has the potential of reestablishing tissue homeostasis and turning on as a result the immune cancer kill switch.

## Conclusions

Cancer therapies should aim for a progressive disruption of the CCs-TME dynamics and target metabolic dysregulation and inflammation to partially restore tissue homeostasis and turn on the immune cancer kill switch. One potentially effective cancer therapeutic strategy is to induce the reduction of lactate and steer the TME to a state of reduced inflammation so as to enable an effective intervention of the immune system. The translation of this therapeutic approach into treatment regimens would however require more understanding of the adaptive complexity of cancer resulting from the interactions of cancer cells with the tumor microenvironment and the immune system.

## Additional file

Additional file 1: Tables S1-S3. Estimated dysregulation probabilities of cellular processes, cardinal pathways and select genes by cancer type. (XLSX 28 kb)

## Abbreviations

CAF: Cancer associated fibroblast
PPP: Pentose phosphate pathway
TAM: Tumor associated macrophages
TCA: Tricarboxylic acid
TME: Tumor microenvironment
